# A Retrospective Cohort Study of the Association between Serum Osteopontin Levels and Aortic Stiffness in Hypertensive Patients

**DOI:** 10.3390/ijerph19010477

**Published:** 2022-01-02

**Authors:** Yuan-Chieh Chang, Jen-Pi Tsai, Ji-Hung Wang, Bang-Gee Hsu

**Affiliations:** 1 Department of Internal Medicine, Hualien Tzu Chi Hospital, Buddhist Tzu Chi Medical Foundation, Hualien 97004, Taiwan; alleneinstein0411@tzuchi.com.tw; 2 Division of Nephrology, Department of Internal Medicine, Dalin Tzu Chi Hospital, Buddhist Tzu Chi Medical Foundation, Chiayi 62247, Taiwan; tsaininimd1491@gmail.com; 3 School of Medicine, Tzu Chi University, Hualien 97004, Taiwan; 4 Division of Cardiology, Hualien Tzu Chi Hospital, Buddhist Tzu Chi Medical Foundation, Hualien 97004, Taiwan; 5 Division of Nephrology, Hualien Tzu Chi Hospital, Buddhist Tzu Chi Medical Foundation, Hualien 97004, Taiwan

**Keywords:** osteopontin, aortic stiffness, hypertension, carotid–femoral pulse wave velocity

## Abstract

By suppressing mineralization and preventing ectopic calcium deposits, osteopontin (OPN) has an inhibitory effect on vascular calcification. Also, there is an association between OPN and aortic stiffness (AS). We aimed to investigate the association between serum OPN levels and AS measured by carotid–femoral pulse wave velocity (cfPWV) in hypertensive patients. Baseline characteristics and fasting blood sampling of 120 participants with hypertension and 120 participants without hypertension were acquired. Serum OPN concentrations were determined by enzyme-linked immunosorbent assay. In total, 43 (35.9%) participants were assigned to the AS group with cfPWV of >10 m/s in hypertensive patients. There were more patients with diabetes mellitus, old age, high systolic blood pressure, high serum intact parathyroid hormone (iPTH), elevated C-reactive protein, and high OPN levels in the AS group compared with the control group in hypertensive participants. A multivariate logistic regression analysis discloses that age, SBP, serum OPN, and iPTH levels were independently associated with AS in hypertensive patients. Moreover, according to a multivariate forward stepwise linear regression analysis, OPN level is positively associated with cfPWV. In conclusion, serum OPN level is assumed to be a potential biomarker to predict AS and is positively associated with cfPWV in patients with hypertension.

## 1. Introduction

Hypertension (HTN) is correlated with future cardiovascular disease (CVD) events. Treated but uncontrolled hypertensive patients and untreated hypertensive patients are all at increased risk of all-cause and CVD-related mortality [1]. The association between cardiovascular risk and demographic and laboratory risk factors, including smoking, diabetes, age, sex, and hypercholesterolemia, has been well assessed [2]. Arterial stiffness is also a predictive factor of CVD events in some studies [3,4]. A large, exploratory, post hoc analysis revealed that the markers of aortic stiffness (AS), including estimated pulse wave velocity (PWV), can be a predictor of CV risk. Moreover, they might be effective treatment targets [5]. Smoking, HTN, diabetes mellitus (DM), and hypercholesterolemia, which are associated with early arterial stiffening, are also CVD risk factors of HTN [2,6]. AS is closely associated with HTN, and both are risk factors of future CVD events [7].

Osteopontin (OPN) is produced by cells involved in bone morphogenesis, and is a highly phosphorylated glycophosphoprotein with multiple functions. Moreover, it plays an important role in CVD, inflammation, kidney stone disease, and biomineralization [8]. OPN may inhibit vascular calcification and promote dissolution with consideration of critical factors such as phosphorylation and the presence of certain isoforms [8,9]. In coronary artery disease, serum OPN level correlated positively with AS by measuring carotid–femoral pulse wave velocity (cfPWV) [10]. However, in hypertensive patients, studies evaluating OPN and AS by measuring cfPWV values are limited. Thus, the current study was aimed at investigating the association between OPN and AS in hypertensive patients.

## 2. Materials and Methods

### 2.1. Participants

From August 2020 to December 2020, with the approval of the research ethics committee at a medical center in Taiwan, 138 participants with HTN in the CV outpatient department were recruited, and written informed consent from all participants was obtained. Of these HTN participants, 18 participants were excluded because of heart failure (*n* = 3), malignancy (*n* = 5), acute myocardial infarction (*n* = 1), acute phase of infection (*n* = 3), and refusal to provide informed consent (*n* = 6). In total, 120 participants with HTN were included in the research. Another 120 participants without HTN were also included in this study. Among 240 participants, 169 were men and 71 women. All participants were required to rest for at least 10 min in the morning before having their blood pressure measured with standard mercury sphygmomanometers by trained staff. The staff recorded systolic blood pressure (SBP) when Korotkoff sounds were absent, and diastolic blood pressure (DBP) when Korotkoff sounds were present. The average value was used in the analysis. The definition of HTN was an SBP of ≥140 mmHg and/or DBP of ≥90 mmHg, or having taken anti-hypertensive drugs within the last 2 weeks.

### 2.2. Body Height, Body Weight, Body Mass Index Measurements

Body height (BH) and body weight (BW) were measured with bare feet and light clothing. Body mass index (BMI) was calculated as BW (kg)/BH-squared (m^2^) [11,12].

### 2.3. Measurement of Biochemical Data, Intact Parathyroid Hormone, and Osteopontin

About 5 c.c. fasting blood samples were collected and then centrifuged for 10 min at 3000 *g* without any delay, to measure baseline biochemical data including blood urea nitrogen (BUN), creatinine, fasting glucose, total cholesterol, triglyceride (TG), high-density lipoprotein cholesterol (HDL-C), low-density lipoprotein cholesterol (LDL-C), total calcium, phosphorus, and C-reactive protein (CRP) levels by an autoanalyzer (Siemens Advia 1800; Siemens Healthcare GmbH, Henkestr, Germany) [11,12]. We used enzyme-linked immunosorbent assays to assess serum intact parathyroid hormone (iPTH) levels (Abcam, Cambridge, MA, USA) and human OPN levels (Thermo Fisher Scientific Inc., Waltham, MA, USA) [13,14]. CKD-EPI (Chronic Kidney Disease Epidemiology Collaboration) equation was used to assess estimated glomerular filtration rate (eGFR).

### 2.4. Measurement of Carotid–Femoral Pulse Wave Velocity

In a quiet and temperature-controlled room, participants were in supine position and resting for more than 10 min before cfPWV measurement was obtained by using a pressure tonometer (SphygmoCor System, AtCor Medical, Sydney, New South Wales, Australia) [11,12]. We used the integral software to process the cfPWV data. We also set quality indices using the software, ensuring data uniformity. HTN participants were then divided into the AS group and control group by cfPWV values more than 10 m/s or ≤10 m/s, respectively [2,15].

### 2.5. Statistical Analysis

Kolmogorov–Smirnov test was used to examine the normality of baseline continuous variables. Data expressed as normal or non-normal distribution were expressed as mean ± standard deviation or median and interquartile ranges; comparisons between groups were analyzed by two-tailed Student’s independent t-test or Mann–Whitney U test. Categorical variables were analyzed by using the χ2 test. Multivariate logistic regression analysis was used for the variables significantly associated with AS. Fasting glucose, BUN, creatinine, TG, HDL-C, CRP, and iPTH levels were not in normal distribution, so we performed base 10 logarithmic transformations of those data, achieving normality. We then used a simple linear regression and multivariable forward stepwise regression analysis to assess the correlation between clinical variables and cfPWV or OPN levels in hypertensive patients. The area under the receiver operating characteristic curve was calculated to identify the optimal OPN levels associated with AS. The Statistical Package for the Social Sciences software for Windows (version 19.0; SPSS, Chicago, IL, USA) was used for data analysis. A *p* value of <0.05 was considered statistically significant.

## 3. Results

### 3.1. The Baseline Variables Compared between Patients with or without Hypertension

The baseline demographic and biochemical characteristics of participants with or without hypertension are shown in Table 1. Compared with participants without hypertension, hypertensive participants had higher percentages of coronary artery disease (*p* < 0.001), higher SBP (*p* < 0.001), and higher cfPWV values (*p* = 0.044).

### 3.2. The Baseline Variables Compared between the AS and Control Groups in Participants with Hypertension

The baseline demographic and biochemical characteristics and medications of participants with HTN used are shown in Table 2. In total, there were 43 (35.8%) hypertensive participants in the AS group. Compared with the control group, hypertensive participants in the AS group were older (*p* < 0.001) and had higher SBP (*p* = 0.019), serum iPTH (*p* = 0.003), CRP (*p* = 0.023), and OPN (*p* < 0.001) levels. A higher percentage of DM (*p* = 0.039) was also noted in the AS group compared with the control group. However, there were no statistically significant differences in terms of gender and the usage of medications between these two groups in participants with hypertension.

### 3.3. Variables Associated with Aortic Stiffness in Participants with Hypertension

**Figure 1:**
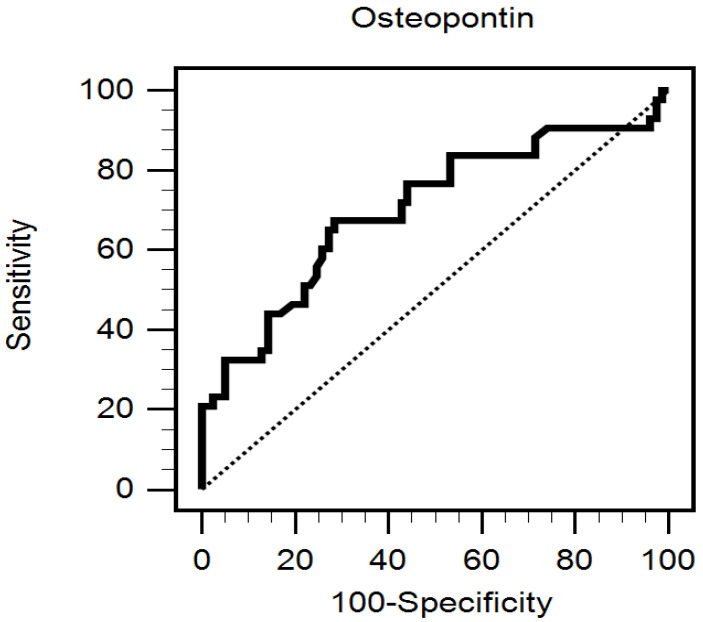
Receiver operating characteristic curve of osteopontin on the prediction of aortic stiffness in hypertensive patients.

By forward multivariate logistic regression analysis adjusted data for factors including eGFR, DM, SBP, and age and CRP, iPTH, and OPN levels, it was revealed that the variables independently associated with AS in hypertensive participants were OPN (odds ratio [OR] = 1.053; 95% confidence interval [CI] = 1.018–1.089; *p* = 0.003), age (OR =1.108, 95% CI = 1.037–1.184, *p* = 0.002), iPTH level (OR = 1.031, 95% CI = 1.011–1.051, *p* = 0.002), and SBP (OR = 1.034, 95% CI = 1.002–1.067, *p* = 0.037) (Table 3). By ROC curve analysis, the area under the receiver operating characteristic curve of OPN level was 0.703 (95% CI = 0.613–0.783, *p* = 0.0001), with a sensitivity and specificity of 67.44% and 71.43%, respectively (Figure 1).

### 3.4. Correlation between cfPWV and Clinical Variables in Participants with Hypertension

Table 4 depicts the correlation between clinical variables and cfPWV in hypertensive participants. A simple linear regression analysis revealed that DM (*r* = 0.186, *p* = 0.042), SBP (*r* = 0.338, *p* = 0.001), and age (*r* = 0.355, *p* < 0.001), and log-CRP (*r* = 0.249, *p* = 0.006), log-BUN (*r* = 0.246, *p* = 0.007), log-creatinine (*r* = 0.348, *p* < 0.001), log-iPTH (*r* = 0.297, *p* = 0.001), and OPN (*r* = 0.536, *p* < 0.001) were positively correlated with cfPWV. Meanwhile, eGFR (*r* = −0.282, *p* = 0.002) was negatively associated with cfPWV. After adjusting for significant factors, including age, DM, SBP, eGFR, and log-BUN, log-creatinine, log-iPTH, log-CRP, and OPN levels, the forward multivariate stepwise linear regression analysis showed that DM (*p* = 0.022), age (*p* = 0.006), SBP (*p* < 0.001), log-iPTH (*p* < 0.001), and OPN (*p* < 0.001) were significantly correlated with cfPWV in patients with hypertension.

### 3.5. Correlation between Osteopontin and Clinically Significant Variables in Participants with Hypertension

Table 5 depicts the correlation between clinically significant variables and serum OPN levels in hypertensive participants. A simple linear regression analysis revealed that hypertensive patients with coronary artery disease (*r* = 0.216, *p* = 0.018), age (*r* = 0.252, *p* < 0.005) and SBP (*r* = 0.191, *p* = 0.037) were positively correlated with OPN levels. Meanwhile, eGFR (*r* = −0.254, *p* = 0.005) was negatively associated with OPN levels. After adjusting for significant factors, including coronary artery disease, age, SBP, and eGFR, the forward multivariate stepwise linear regression analysis showed that coronary artery disease (β = 0.208, adjusted R^2^ change = 0.035, *p* = 0.019) and eGFR (β = −0.247, adjusted R^2^ change = 0.057, *p* = 0.006) were significantly correlated with OPN levels in patients with hypertension.

## 4. Discussion

This research primarily noted that hypertensive patients had higher cfPWV values than participants without hypertension. cfPWV values were associated with old age, DM, SBP, iPTH, and OPN levels in hypertensive patients. In addition, serum OPN level, old age, SBP, and CRP level were the independent predictors for the development of AS in this patient group.

AS, which is attributed to aging, inflammation, reactive oxygen species production, vascular calcification, elastin fiber degradation, and collagen deposition in aortic walls cause increased pulse wave velocity. This then damages the heart by increasing the cardiac workload and by reducing coronary artery perfusion pressure. Further, AS is associated with all-cause mortality and CVD [7,16,17]. Several risk factors, such as aging, high SBP, DM, metabolic syndrome, inflammation, and chronic renal failure contribute to the development and progression of AS [7,17]. Aging enhances the severity of vascular damage in HTN [18]. In DM, endothelial nitric oxide dysregulation and a high expression of advanced glycation end-products play critical roles for the vascular damage in these patients [19]. A previous meta-analysis study of 11,781 patients has shown that SBP is associated with AS progression [20]. In hypertensive patients, CRP level is positively corrected with AS and can predict future CVD [21]. Similarly, cfPWV is positively associated with DM, age, SBP, and CRP level in hypertensive patients. Moreover, a higher incidence of DM, higher SBP, and older age were found in patients with HTN who presented with AS after adjusting for covariates.

AS can increase pulsatile pressure and pulsatile flow, which then causes microvascular damage in the kidney [16]. In patients with HTN, cfPWV is negatively associated with eGFR independently of other confounding factors [22]. Cheng et al. showed that cfPWV independently increased parathyroid hormone levels in 1052 Chinese patients [23]. In addition, Lee et al. found that parathyroid hormone concentration is positively correlated with brachial–ankle baPWV, in a study of 8217 participants in Korea [24]. In hemodialysis patients with secondary hyperparathyroidism, parathyroidectomy reduced baPWV and coronary artery calcification score at 1-year postoperative follow-up [25]. Our study noted that log-iPTH level was positively correlated, and eGFR was negatively correlated with cfPWV in hypertensive patients. Serum iPTH level was significantly associated with AS after adjusting for several confounders.

Vascular calcification is also a risk factor of AS [7,26]. OPN hinders the formation of hydroxyapatite crystals, and play a potent role in vascular calcification inhibition [26]. A higher OPN level had a counter-regulatory process that further decreased ossification and calcium accumulation in the arterial system of vascular calcification [27]. In an animal study, OPN deficiency increased vascular endochondral mineralization, chondroid metaplasia, and collagen accumulation with progression in LDLR−/− mice [28]. In human studies, serum OPN level is positively associated with cfPWV in geriatric adults, healthy participants, coronary artery disease, and those who received kidney transplantation [10,13,14,29]. In a study of type 2 DM, Sharif et al. revealed a positive correlation between plasma OPN level and a high arterial stiffness summary score [30]. Taken together, our results showed that OPN level was positively associated with cfPWV after a multivariate forward stepwise linear regression analysis. After adjusting for confounding factors, a higher serum OPN level was found to be an independent predictor of AS in patients with hypertension.

The association between OPN and impaired nitric oxide (NO) bioavailability could play a role in the inhibition of endothelial NO synthase in coronary artery disease patients [31]. Serum OPN identifies progression of coronary artery calcium scanning in patients without known atherosclerotic cardiovascular disease at baseline after 4-year follow-up [32]. Many studies also noted serum OPN levels were negatively associated with eGFR [33,34]. Our results also noted hypertensive patients with coronary artery disease are positively associated, while eGFR is negatively associated, with serum OPN levels after confounder adjustment in patients with hypertension.

Several limitations were found in the current study. First, the study was cross-sectionally designed with a limited number of participants. Hence, we cannot exclude the risk of indication bias. Second, OPN phosphorylation is required to inhibit vascular smooth muscle cell calcification [35]. However, the nowadays available OPN assays cannot distinguish phosphorylated types from non-phosphorylated types. Third, β-blockers such as nebivolol and bisoprolol, which are anti-hypertensive drugs, can influence cfPWV [36,37]. However, our results did not confirm the relationship between anti-hypertensive medication and AS. Thus, in patients with HTN, further studies are required to confirm the association between OPN level and AS.

## 5. Conclusions

Our results noted higher serum OPN level is associated with cfPWV values and is independently associated with AS in hypertensive patients. Nevertheless, in hypertensive patients, follow-up studies should be performed to investigate the association of serum OPN level and AS.

## Figures and Tables

**Table 1:** Clinical and analytical characteristics of patients with or without hypertension.

Variables	All Participants (*n* = 240)	Non-Hypertension (*n* = 120)	Hypertension (*n* = 120)	*p* Value
Age (years)	64.26 ± 8.53	63.65 ± 7.11	64.87 ± 9.74	0.269
Height (cm)	162.08 ± 7.62	162.84 ± 6.93	161.32 ± 8.21	0.121
BW (Kg)	68.95 ± 11.53	68.86 ± 10.84	69.04 ± 12.23	0.905
BMI (Kg/m^2^)	26.22 ± 3.84	26.01 ± 4.13	26.43 ± 3.54	0.397
cfPWV (m/s)	9.00 ± 2.49	8.68 ± 2.25	9.33 ± 2.68	0.044 *
SBP (mmHg)	128.00 (120.00–135.00)	123.50 (116.00–130.00)	134.00 (126.00–146.75)	<0.001 *
DBP (mmHg)	73.89 ± 9.50	73.57 ± 8.41	74.22 ± 10.50	0.597
Total cholesterol (mg/dL)	170.48 ± 35.57	167.37 ± 29.62	173.60 ± 40.54	0.175
TG (mg/dL)	122.00 (88.00–169.75)	121.50 (87.00–173.75)	122.00 (89.25–167.50)	0.704
HDL-C (mg/dL)	44.00 (38.00–54.00)	45.00 (38.00–55.75)	42.00 (37.25–54.00)	0.389
LDL-C (mg/dL)	99.37 ± 26.94	96.68 ± 22.64	102.06 ± 30.50	0.122
Fasting glucose (mg/dL)	112.00 (96.00–147.00)	110.00 (95.00–143.25)	115.50 (97.00–160.50)	0.101
BUN (mg/dL)	16.00 (13.00–18.75)	16.00 (13.00–18.00)	16.00 (13.00–19.00)	0.222
Creatinine (mg/dL)	1.05 (0.90–1.20)	1.05 (1.00–1.20)	1.05 (0.90–1.30)	0.937
eGFR (mL/min)	72.73 ± 16.92	74.29 ± 10.43	71.16 ± 21.48	0.153
Total calcium (mg/dL)	9.13 ± 0.36	9.11 ± 0.35	9.16 ± 0.37	0.243
Phosphorus (mg/dL)	3.49 ± 0.51	3.49 ± 0.52	3.49 ± 0.50	0.950
iPTH (pg/mL)	44.50 (33.28–63.95)	44.45 (33.70–61.63)	45.55 (32.98–64.85)	0.790
CRP (mg/dL)	0.21 (0.15–0.28)	0.20 (0.15–0.27)	0.21 (0.15–0.29)	0.358
Osteopontin (pg/mL)	27.67 ± 16.45	27.22 ± 14.43	28.12 ± 18.31	0.676
Female, *n* (%)	71 (29.6)	33 (27.5)	38 (31.7)	0.479
DM, *n* (%)	80 (33.3)	36 (30.0)	44 (36.7)	0.273
CAD, *n* (%)	112 (46.7)	30 (25.0)	82 (68.3)	<0.001 *

Values for continuous variables are given as means ± standard deviations or medians and interquartile ranges; categorical variables are expressed as numbers (%). Abbreviations: BW, body weight; BMI, body mass index; cfPWV, carotid–femoral pulse wave velocity; SBP, systolic blood pressure; DBP, diastolic blood pressure; TG, triglyceride; BUN, blood urea nitrogen; iPTH, intact parathyroid hormone; CRP, C-reactive protein; DM, diabetes mellitus; HDL-C, high-density lipoprotein cholesterol; LDL-C, low-density lipoprotein cholesterol; eGFR, estimated glomerular filtration rate; CAD, coronary artery disease. * *p* < 0.05 was considered statistically significant.

**Table 2:** Comparisons of clinical characteristics between aortic stiffness and control group.

Variables	Control Group (*n* = 77)	Aortic Stiffness Group (*n* = 43)	*p* Value
Age (years)	62.34 ± 9.50	69.40 ± 8.53	<0.001 *
Height (cm)	161.71 ± 8.29	160.60 ± 8.12	0.480
BW (Kg)	68.61 ± 13.10	69.81 ± 10.58	0.607
BMI (Kg/m^2^)	26.08 ± 3.54	27.05 ± 3.49	0.150
cfPWV (m/s)	7.77 ± 1.41	12.12 ± 2.08	<0.001 *
SBP (mmHg)	133.49 ± 14.56	140.98 ± 19.70	0.019 *
DBP (mmHg)	74.65 ± 10.36	73.44 ± 10.81	0.548
Total cholesterol (mg/dL)	175.62 ± 41.55	169.98 ± 38.89	0.467
TG (mg/dL)	114.00 (89.00–166.00)	130.00 (91.00–169.00)	0.801
HDL-C (mg/dL)	47.00 (38.00–54.00)	41.00 (36.00–54.00)	0.285
LDL-C (mg/dL)	102.65 ± 29.46	101.00 ± 32.60	0.778
Fasting glucose (mg/dL)	115.00 (97.50–165.50)	116.00 (96.00–157.00)	0.928
BUN (mg/dL)	15.00 (13.00–19.00)	17.00 (14.00–22.00)	0.056
Creatinine (mg/dL)	1.00 (0.90–1.20)	1.20 (0.90–1.40)	0.057
eGFR (mL/min)	73.63 ± 19.62	66.74 ± 24.07	0.092
Total calcium (mg/dL)	9.18 ± 0.38	9.13 ± 0.36	0.525
Phosphorus (mg/dL)	3.53 ± 0.51	3.42 ± 0.49	0.778
iPTH (pg/mL)	40.40 (32.25–54.60)	55.00 (40.60–78.90)	0.001 *
CRP (mg/dL)	0.19 (0.12–0.30)	0.24 (0.18–0.29)	0.023 *
Osteopontin (pg/mL)	23.02 ± 13.92	37.25 ± 21.58	<0.001 *
Female, *n* (%)	27 (35.1)	11 (25.6)	0.284
DM, *n* (%)	23 (29.9)	21 (48.8)	0.039 *
CAD, *n* (%)	51 (66.2)	31 (72.1)	0.508
ACE inhibitor, *n* (%)	21 (27.3)	15 (34.9)	0.383
ARB, *n* (%)	48 (62.3)	24 (55.8)	0.484
β-blocker, *n* (%)	41 (53.2)	25 (58.1)	0.605
Calcium channel blocker, *n* (%)	30 (39.0)	21 (48.8)	0.294
Statin, *n* (%)	43 (55.8)	19 (44.2)	0.220
Fibrate, *n* (%)	12 (15.6)	9 (20.9)	0.460

Values for continuous variables are given as means ± standard deviations or medians and interquartile ranges; categorical variables are expressed as numbers (%). Abbreviations: BW, body weight; BMI, body mass index; cfPWV, carotid-femoral pulse wave velocity; SBP, systolic blood pressure; DBP, diastolic blood pressure; TG, triglyceride; HDL-C, high-density lipoprotein cholesterol; LDL-C, low-density lipoprotein cholesterol; BUN, blood urea nitrogen; eGFR, estimated glomerular filtration rate; iPTH, intact parathyroid hormone; CRP, C-reactive protein; DM, diabetes mellitus; CAD, coronary artery disease; ACE, angiotensin-converting enzyme; ARB, angiotensin-receptor blocker. * *p* < 0.05 was considered statistically significant.

**Table 3:** Variables associated with aortic stiffness by multivariable logistic regression analysis in hypertension.

Variables	Odds Ratio	95% Confidence Interval	*p* Value
Osteopontin, 1 pg/mL	1.053	1.018−1.089	0.003 *
Age, 1 year	1.108	1.037−1.184	0.002 *
Intact parathyroid hormone, 1 pg/mL	1.031	1.011−1.051	0.002 *
Systolic blood pressure, 1 mmHg	1.034	1.002−1.067	0.037 *
Diabetes mellitus, present	2.522	0.952−6.676	0.063
C-reactive protein, 1 mg/dL	1.014	0.281−3.653	0.983
eGFR, 1 mL/min	1.016	0.989−1.043	0.252

Multivariate logistic regression analysis was applied to process the data. (Taken factors: diabetes mellitus, age, systolic blood pressure, intact parathyroid hormone, C-reactive protein, eGFR, and osteopontin). Abbreviations: eGFR, estimated glomerular filtration rate. We considered * *p* < 0.05 was statistically significant.

**Table 4:** Correlation between carotid-femoral pulse wave velocity levels and clinical variables in hypertension.

Variables	Carotid-Femoral Pulse Wave Velocity (m/s)				
	Simple Regression		Multivariate Regression		
	*r*	*p* Value	Beta	Adjusted R^2^ Change	*p* Value
Female	−0.172	0.061	–	–	–
DM	0.186	0.042 *	0.161	0.021	0.022 *
Coronary artery disease	0.040	0.662	–	–	–
Age (years)	0.355	<0.001 *	0.196	0.035	0.006 *
BH (cm)	0.012	0.894	–	–	–
BW (Kg)	0.040	0.665	–	–	–
BMI (Kg/m^2^)	0.056	0.545	–	–	–
SBP (mmHg)	0.338	<0.001 *	0.253	0.066	<0.001 *
DBP (mmHg)	0.064	0.485	–	–	–
Total cholesterol (mg/dL)	−0.080	0.383	–	–	–
Log-TG (mg/dL)	0.038	0.683	–	–	–
Log-HDL-C (mg/dL)	−0.153	0.095	–	–	–
LDL-C (mg/dL)	−0.002	0.981	–	–	–
Log-Glucose (mg/dL)	−0.002	0.985	–	–	–
Log-BUN (mg/dL)	0.246	0.007 *	–	–	–
Log-Creatinine (mg/dL)	0.348	<0.001 *	–	–	–
eGFR (mL/min)	−0.282	0.002 *	–	–	–
Total calcium (mg/dL)	0.008	0.932	–	–	–
Phosphorus (mg/dL)	−0.114	0.213	–	–	–
Log-iPTH (pg/mL)	0.297	0.001 *	0.283	0.059	<0.001 *
Log-CRP (mg/dL)	0.249	0.006 *	–	–	–
Osteopontin (pg/mL)	0.536	<0.001 *	0.401	0.281	<0.001 *

Triglyceride, glucose, iPTH, and osteopontin levels were log-transformed before analysis because of skewed distribution. Simple and multivariable stepwise linear regression analyses (adopted factors: diabetes mellitus, age, systolic blood pressure, log-BUN, log-Creatinine, eGFR, log-iPTH. log-CRP, and osteopontin) were applied. Abbreviations: BH, body height; BW, body weight; BMI, body mass index; SBP, systolic blood pressure; DBP, diastolic blood pressure; TG, triglyceride; HDL-C, high-density lipoprotein cholesterol; LDL-C, low-density lipoprotein cholesterol; BUN, blood urea nitrogen; eGFR, estimated glomerular filtration rate; iPTH, intact parathyroid hormone; CRP, C-reactive protein. * *p* < 0.05 was considered statistically significant.

**Table 5:** Correlation between serum osteopontin levels and clinically significant variables in hypertension.

Variables	Osteopontin (pg/mL)				
	Simple Regression		Multivariate Regression		
	*r*	*p* Value	Beta	Adjusted R^2^ Change	*p* Value
Coronary artery disease	0.216	0.018 *	0.208	0.035	0.019 *
Age (years)	0.252	0.005 *	−	−	−
Systolic blood pressure (mmHg)	0.191	0.037 *	−	−	−
eGFR (mL/min)	−0.254	0.005 *	−0.247	0.057	0.006 *

Analysis of data was done using univariable linear regression analyses or multivariable stepwise linear regression analysis (adopted factors: coronary artery disease, age, systolic blood pressure, and eGFR). Abbreviations: eGFR, estimated glomerular filtration rate. * *p* < 0.05 was considered statistically significant.

## Data Availability

The data presented in this study are available on request from the corresponding author.
